# Towards Endometriosis Diagnosis by Gadofosveset-Trisodium Enhanced Magnetic Resonance Imaging

**DOI:** 10.1371/journal.pone.0033241

**Published:** 2012-03-22

**Authors:** Marc H. Schreinemacher, Walter H. Backes, Jos M. Slenter, Sofia Xanthoulea, Bert Delvoux, Larissa van Winden, Regina G. Beets-Tan, Johannes L. H. Evers, Gerard A. J. Dunselman, Andrea Romano

**Affiliations:** 1 GROW – School for Oncology and Developmental Biology, Maastricht University and Medical Centre, Maastricht, The Netherlands; 2 Department of Obstetrics and Gynaecology, Maastricht University and Medical Centre, Maastricht, The Netherlands; 3 Department of Surgery, Maastricht University and Medical Centre, Maastricht, The Netherlands; 4 Department of Radiology, Maastricht University and Medical Centre, Maastricht, The Netherlands; 5 Cardiovascular Research Institute Maastricht, Maastricht University and Medical Centre, Maastricht, The Netherlands; University of Texas, M.D. Anderson Cancer Center, United States of America

## Abstract

Endometriosis is defined as the presence of endometrial tissue outside the uterus. It affects 10–15% of women during reproductive age and has a big personal and social impact due to chronic pelvic pain, subfertility, loss of work-hours and medical costs. Such conditions are exacerbated by the fact that the correct diagnosis is made as late as 8–11 years after symptom presentation. This is due to the lack of a reliable non-invasive diagnostic test and the fact that the reference diagnostic standard is laparoscopy (invasive, expensive and not without risks). High-molecular weight gadofosveset-trisodium is used as contrast agent in Magnetic Resonance Imaging (MRI). Since it extravasates from hyperpermeable vessels more easily than from mature blood vessels, this contrast agent detects angiogenesis efficiently. Endometriosis has high angiogenic activity. Therefore, we have tested the possibility to detect endometriosis non-invasively using Dynamic Contrast-Enhanced MRI (DCE-MRI) and gadofosveset-trisodium as a contrast agent in a mouse model. Endometriotic lesions were surgically induced in nine mice by autologous transplantation. Three weeks after lesion induction, mice were scanned by DCE-MRI. Dynamic image analysis showed that the rates of uptake (inwash), persistence and outwash of the contrast agent were different between endometriosis and control tissues (large blood vessels and back muscle). Due to the extensive angiogenesis in induced lesions, the contrast agent persisted longer in endometriotic than control tissues, thus enhancing the MRI signal intensity. DCE-MRI was repeated five weeks after lesion induction, and contrast enhancement was similar to that observed three weeks after endometriosis induction. The endothelial-cell marker CD31 and the pericyte marker α-smooth-muscle-actin (mature vessels) were detected with immunohistochemistry and confirmed that endometriotic lesions had significantly higher prevalence of new vessels (CD31 only positive) than the uterus and control tissues. The diagnostic value of gadofosveset-trisodium to detect endometriosis should be tested in human settings.

## Introduction

Endometriosis is defined as the presence of endometrial tissue outside the uterus. It affects 10–15% of women during reproductive age and has a big personal and social impact due to chronic pelvic pain, morbidity, subfertility and high health-care costs [1]–[4]. Endometriosis presents as superficial peritoneal implants, as lesions located on the ovaries (endometriomas) or as deeply invasive nodules, which can infiltrate different organs (rectum, bladder, uterus) compromising their functionality [1], [3].

Despite the fact that important progress has been made in the genetic, the molecular and the hormonal mechanisms underlying its pathogenesis [5]–[10], one of the major clinical issues in the management of this disorder is the diagnosis. Initially based on symptoms, medical history and physical examination, the correct diagnosis is delayed for up to 8–11 years after the first complaints have been reported. This is due to the fact that symptoms are similar to other urogenital disturbances and that a non-invasive diagnostic test is lacking [3], [11], [12]. Ultrasound and magnetic resonance imaging (MRI) help diagnosing endometriomas and deeply invasive lesions [13]. However, peritoneal implants, occurring in most of the patients even in the presence of lesions at other locations, are not detected by these methods [14], [15]. In the end, the reference diagnostic standard is laparoscopy, which is invasive, expensive and not free of risks. Scientists and physicians have therefore recognised that the development of a non-invasive diagnostic tool is a priority in endometriosis research [16].

In the pathogenesis of endometriosis, novel blood vessels are formed [1], [12], [17]–[19]. Angiogenesis can be visualised by Dynamic Contrast-Enhanced MRI (DCE-MRI) using gadolinium-based contrast agents [20]–[24]. However, this technique has shown already little accuracy and a sensitivity lower than 40% in diagnosing peritoneal endometriosis [14], [15], [25].

Recent experience in oncology, has underscored that high-molecular weight contrast agents detect novel blood vessels with higher accuracy, contrast and sensitivity than low-molecular molecules such as gadolinium [26]–[28]. Gadofosveset-trisodium is a gadolinium-based contrast, which forms a complex with albumin in the blood, hence becomes a macromolecule [28]. This complex extravasates easily for immature hyperpermeable blood vessels but not (or much slower) from mature vasculature. As a result, the signal from angiogenic spots is enhanced. Gadofosveset-trisodium is well characterised for angiography and for colorectal cancer diagnosis [20]–[24], [26]–[28]. Its use in diagnosing endometriosis has never been explored. Therefore, we devised an experimental study in which the detection of surgically induced endometriotic lesions in a small-animal model was evaluated by DCE-MRI using gadofosveset-trisodium as a contrast agent. Our results show that endometriosis-associated angiogenesis could be visualised by this method in mice, which implies diagnostic potential in patients in the near future.

## Results

### Endometriotic lesions induction

**Figure 1:**
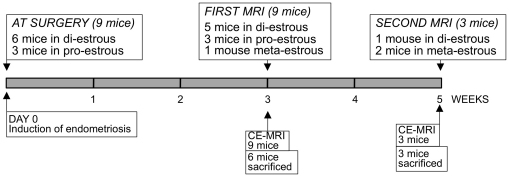
Scheme of the animal experiment. Endometriosis was surgically induced by autologous transplantation [29] in nine mice at DAY 0. Three weeks (all nine mice) and five weeks later (three mice), animals were subjected to DCE-MRI.

**Figure 2:**
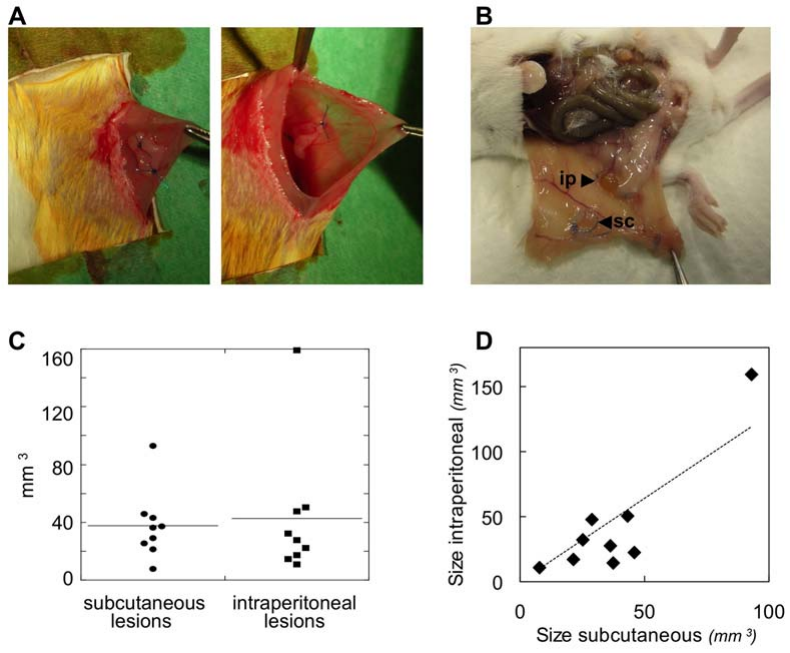
Endometriosis was induced in mice. A. Autologous transplantation [29]: one uterine horn from one mouse is removed, opened longitudinally, and two 2–3 mm^2^ uterine fragments are sutured to the abdominal wall of the same mouse either subcutaneously (left) or intraperitoneally (right). B. At sacrifice, macroscopic inspection revealed the presence of both subcutaneous (sc) and intraperitoneal (ip) lesions. C. No significant size differences were seen between subcutaneous and intraperitoneal lesions. D. The size of the subcutaneous and intraperitoneal lesions in each single mouse are plotted on the X and Y-axes, respectively, and showed a significant correlation (R^2^ = 0.7930).

Lesions were surgically induced by autologous transplantation [29] in nine female mice (two lesions per mouse). At time of surgery, six mice were in the di-estrous phase of the estrous cycle and three, in the pro-estrous phase (scheme in Figure 1). One uterine horn from each mouse was removed and opened longitudinally. Two fragments of about 2–3 mm^2^ were sutured to the abdominal wall of the same mouse at opposite abdomen sites, either subcutaneously or intraperitoneally (Figure 2A).

Six mice were sacrificed three weeks after endometriosis induction (just after MRI scans), whereas three mice were maintained for five weeks after endometriosis induction and were scanned for a second time. At sacrifice, macroscopic inspection revealed the presence of both subcutaneous and intraperitoneal lesions (Figure 2B). No significant size differences were measured between subcutaneously and intraperitoneally induced endometriosis (Figure 2C). In each mouse, the size of the lesions in the two locations was correlated (Figure 2D). No difference was observed among lesions induced at distinct phases of the estrous cycle.

**Figure 3:**
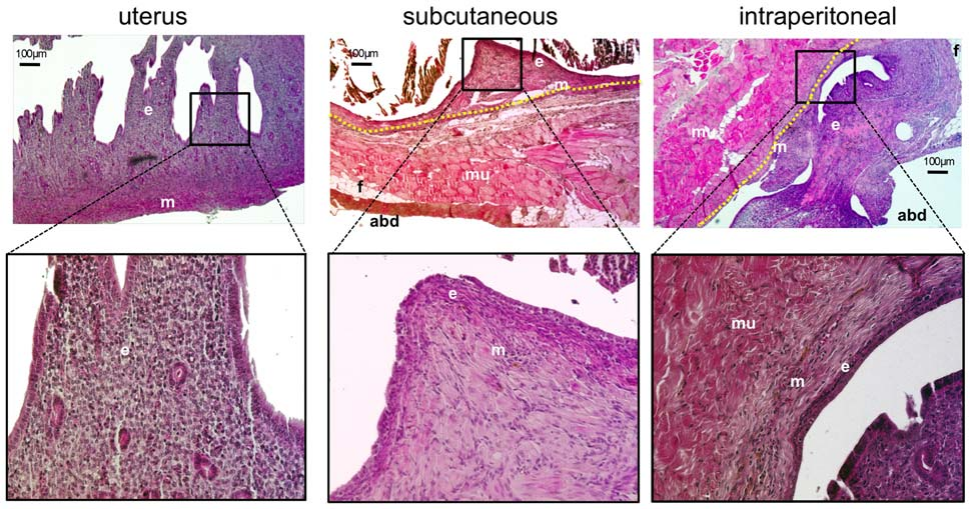
Endometriosis was confirmed with histology. Haematoxylin and eosin staining was used to characterise histologically the lesions. ‘e’ indicates endometrial tissue; ‘m’ = myometrium. Muscle fibres and fat tissue of the abdominal wall are indicated by: ‘mu’ = muscle; ‘f’ = fat; ‘abd’ = abdominal cavity. The border between endometriosis and the abdominal wall is indicated by the yellow-dashed line.

Histology revealed the presence of typical endometriosis, with cystic or with organised endometrial tissue characteristics (Figure 3). No difference in the histology was observed between subcutaneous and intraperitoneal endometriosis.

### Gadofosveset-trisodium enhancement characteristics

**Figure 4:**
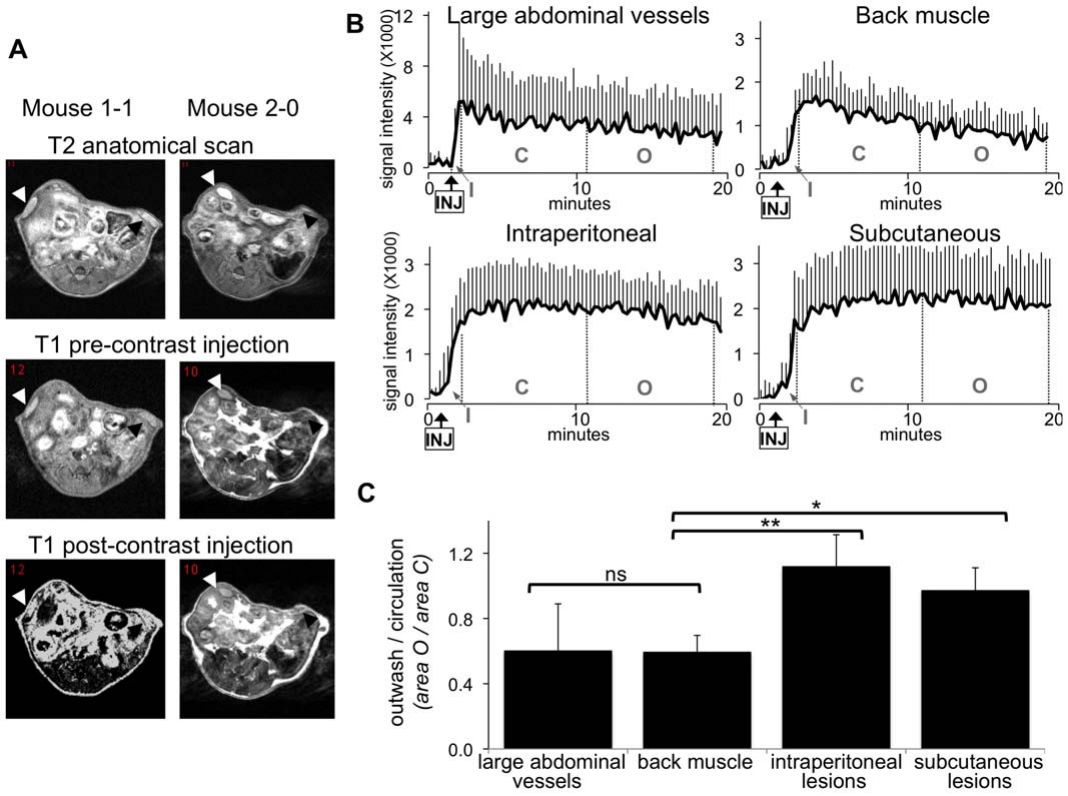
Endometriosis visualised by gadofosveset-trisodium enhanced MRI. Three weeks after lesion induction, mice were subjected to gadofosveset-trisodium enhanced MRI. A. Examples of T2 weighted anatomical images and T1 weighted prior and post contrast injection in two mice. Lesions are indicated by black arrow-heads (subcutaneous) and white arrow-heads (intraperitoneal). B. DCE-MRI derived intensity time-curves in the large abdominal blood vessels, back muscle, intraperitoneal and subcutaneous lesions. Injection of gadofosveset-trisodium is indicated (arrow-INJ). The inwash (I), circulation (C) and the outwash phases (O) of the contrast agent are indicated. Charts represent the average signal intensity of nine mice plus SD. C. The ratio O/C was assessed in the large abdominal vessels, back muscle and endometriosis. Statistic based on unpaired Wilcoxon test: * = 0.001; **<0.001; ns = non-significant.

Three weeks after endometriosis induction, all mice were subjected MRI (scheme in Figure 1). Five mice were in the di-estrous phase, three in the pro-estrous and one in the meta-estrous phase of the cycle. Anatomical T2 and T1 weighted images were acquired prior and post gadofosveset-trisodium administration to visualise the lesions. The induced endometriotic implants could be clearly visualised (Figure 4A). The signal intensity in distinct areas could be subsequently measured through the serial scanned images. After the injection (‘INJ’ in Figure 4B), the contrast almost immediately entered the circulation (inwash phase; ‘I’ in Figure 4B). As expected, the large abdominal vessels (combination of the aorta and vena cava) showed a more rapid enhancement (steeper curve) than the tissues of back muscle and endometriotic lesions, indicating the entrance of the contrast agent in the circulatory system after the injection. The inwash phase was followed by a phase during which the contrast agent steadily circulated (‘C’ in Figure 4B) and finally was cleared during the outwash phase (‘O’ in Figure 4B). In the large abdominal vessels and the back muscle (used as a control of a non-angiogenic tissue) a relatively fast outwash phase was observed, indicated by the decrease of the signal intensity. In the subcutaneous as well as in the intraperitoneal lesions, on the contrary, no signal decrease was observed, indicating that the contrast had extravasated from immature blood vessels into the interstitial space of the surrounding endometriotic tissue, thus enhancing signal MRI intensity.

To quantify the extent of extravasation of the contrast agent, the ratios between washout and inwash (O/C) were computed for the various tissues. The higher values of the O/C ratios in the lesions compared with the control tissues (abdominal vessels and back muscle) indicated that the contrast had extravasated and its presence persisted in induced endometriosis. Contrarily, the low O/C values (control tissues) indicated that the contrast had remained in the blood vessels and washed-out, with little extravasation (Figure 4C).

No difference was observed in the performance of the DCE-MRI in mice at distinct estrous-phase periods. In three mice (one during the di-estrous and two during the meta-estrous phase of the cycle), DCE-MRI was repeated five weeks after lesion induction. No significant differences were seen compared to the DCE-MRI performed two weeks earlier (results not shown).

### Endometriotic lesions surgically induced in mice are highly angiogenic

**Figure 5:**
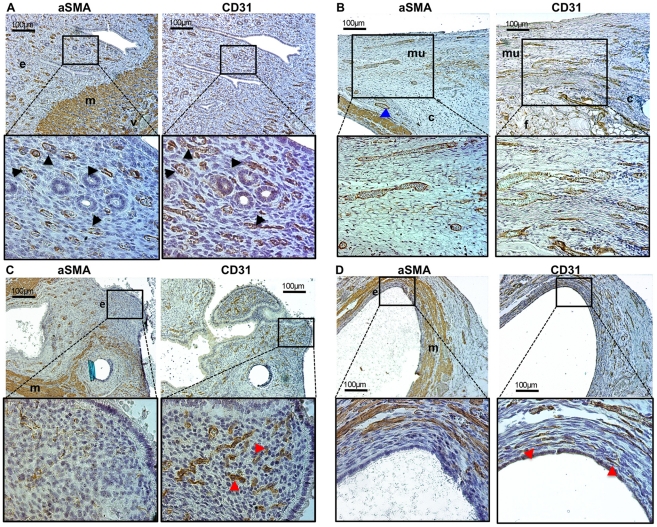
Endometriosis is characterised by angiogenesis. Different tissues were stained by immunohistochemistry for CD31 (endothelial cell marker) and the pericyte marker aSMA (present in mature vessels only). Representative images are shown. A. In the uterus (eutopic endometrium), most CD31 positive blood vessels are also aSMA positive, indicated by the black arrow-heads in the enlargements. ‘e’ = endometrium; ‘m’ = aSMA positive myometrial cells; ‘v’ = large-mature blood vessels present in the myometrial layer. B. Abdominal wall consisting in large part of striated muscular tissue (mu), with some connective (c) and fat (f) tissues. A surgically induced endometriotic lesion is present on the left image, and visible as aSMA positive myometrial tissue (at the bottom-left of the low-magnification image; blue arrow-head). C & D. Intraperitoneal (C) and subcutaneous (D) lesions. In the endometrial layer of the lesions (e), most blood vessels are DC31 only positive (red arrow-heads in the enlargements); ‘m’ = myometrium (aSMA positive cells).

To confirm the presence of angiogenesis in endometriosis, immunohistochemistry was performed in six mice. Cluster of differentiation 31 (CD31) is an endothelial cell marker expressed in all blood vessels, whereas alpha-smooth-muscle-actin (aSMA) [30] is a myofibroblast marker expressed in smooth muscle fibres including pericytes, which are deposited and surround mature vessels [31]. The number of new blood vessels (CD31 only positive) and the number of mature (aSMA positive) blood vessels was assessed in individual tissues (Figure 5).

In the uterus (eutopic endometrium), most CD31 positive blood vessels also expressed aSMA. The myometrium can be easily identified because it consists of aSMA positive fibro muscular cells (Figure 5A). In the (striated and therefore aSMA negative) muscular tissues of the abdominal wall adjacent to the induced endometriosis (peri-endometriotic tissue) most vessels were both CD31 and aSMA positive (Figure 5B). In contrast, in the endometrial layer of both intraperitoneal and subcutaneous lesions (Figure 5C and 5D, respectively), only CD31 positive blood vessels were present, indicating angiogenesis. In this mouse-model, the myometrium was transplanted together with the endometrium, and consisted of aSMA positive cells. This myometrial layer could be easily recognised between the endometrium and the tissues surrounding the lesions (Figure 5B and 5D).

**Figure 6:**
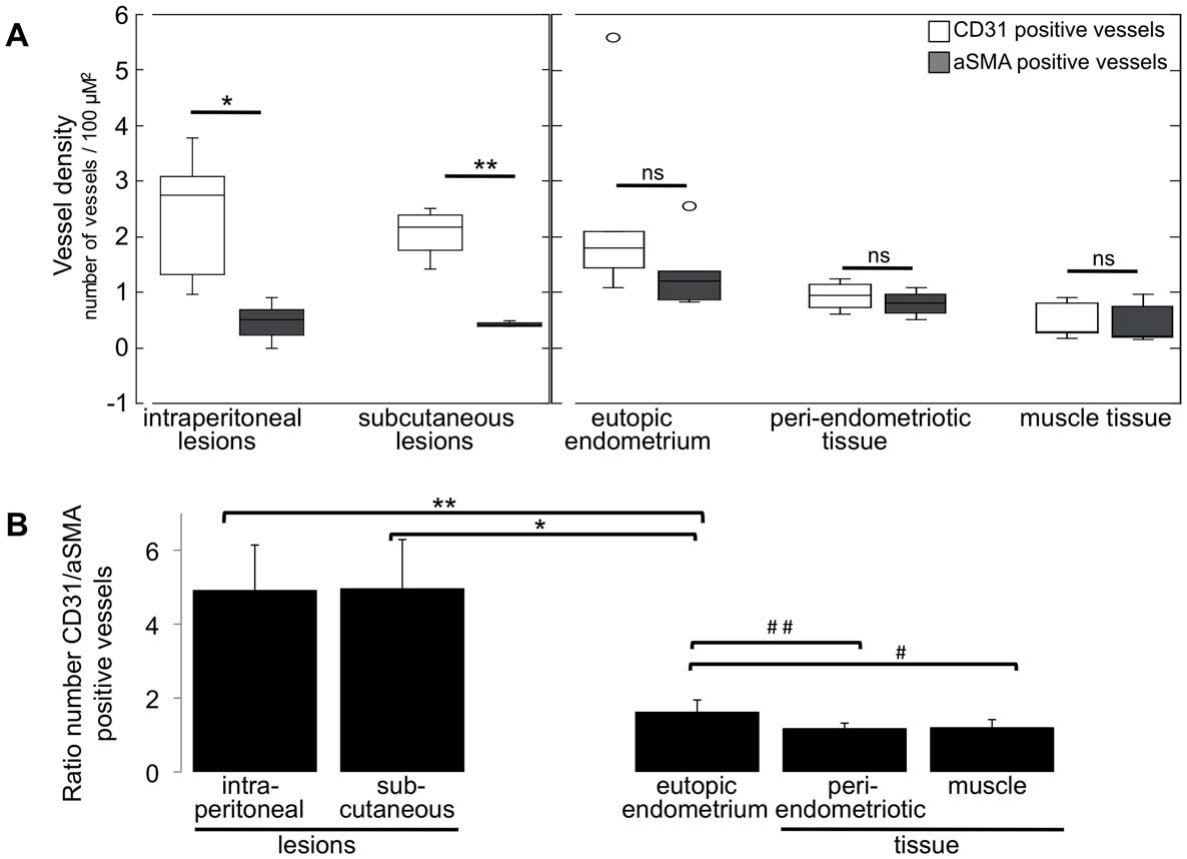
Quantification of new and mature blood vessels. A. The number of CD31 and aSMA positive blood vessels, or vessel density (number vessels per 100 µm^2^) was quantified in: intraperitoneally and subcutaneously induced endometriosis; eutopic endometrium (inside the uterus); muscular tissue of the abdominal wall either adjacent (peri-endometriotic) or distant to the induced lesions (muscle). The average number of vessels in each tissue for six mice is shown; statistic is based on unpaired Wilcoxon test: * = 0.002; ** = 0.029; ns = non-significantly different. B. The ratio between CD31 and aSMA positive blood vessels was calculated in corresponding locations (endometriosis, uterus, peri-endometriosis and muscle) in each mouse. Afterwards, the average CD31/aSMA ratio in each tissue was computed among the six mice. Statistic based on unpaired Wilcoxon test: * = 0.010; ** = 0.004; # = 0.030; ## = 0.019.

Quantification of the number of CD31 and aSMA positive blood vessels in various tissues (vessel density) showed that in both intraperitoneal and subcutaneous endometriosis, the number of CD31 vessels largely exceeded the number of aSMA positive vessels, whereas in the eutopic endometrium (inside the uterus) and the muscular tissue of the abdominal wall adjacent to the endometriosis lesions (peri-endometriotic tissue) or distant from the lesions, equal numbers of CD31 and aSMA positive vessels were observed (Figure 6A). This analysis was performed by assessing independently the number of CD31 and the number of aSMA positive blood vessels per tissue in each mouse and by computing the average vessel density (from the six measured mice) in each tissue (see also material and methods).

In an additionally analysis, to confirm that in each single mouse the surgically induced endometriotic lesions had more CD31 positive vessels than the control tissues, the ratio between the number of CD31 and aSMA positive vessels in each tissue per mouse was determined. Figure 6B illustrates the average CD31/aSMA ratio, which was computed out of the six mice. Endometriotic lesions had about a five-fold increased number of newly formed vessels than the eutopic endometrium. As expected, the endometrium inside the uterus itself had higher angiogenesis than the muscular tissue present in the abdominal wall, as indicated by the increased quantity of newly formed vessels (higher CD31/aSMA ratio) in the (eutopic) endometrium compared with the muscular tissue (Figure 6B).

## Discussion

### Current findings

Gadofosveset-trisodium detects novel blood vessels with high accuracy, contrast and sensitivity [26]–[28] therefore it has been extensively used for angiography and in cancer-associated angiogenesis [20]–[24], [26]–[28].

Angiogenesis has been investigated in endometriosis using mouse models consisting of transplanted murine as well as human endometrial tissues. The formation of novel blood vessels characterises these lesions, and blockade of angiogenesis impairs their growth [1], [12], [17]–[19]. A recent investigation using a mouse model has clearly shown that endothelial progenitor stem cells are increased in mice with induced endometriosis compared with controls, and that these cells are recruited to endometriosis and form neo-vasculature [19].

In the present study, endometriotic lesions were surgically induced in mice at two anatomical locations: intraperitoneally and subcutaneously. Neither difference in size, nor in histological features were observed between intraperitoneal and subcutaneous lesions, indicating that the growth/maintenance of the induced endometriosis was not influenced by the implantation location.

Additionally, using this model, we demonstrated that gadofosveset-trisodium enhanced MRI could detect the induced endometriotic lesions because of the high angiogenic activity of the transplanted endometrium. This was confirmed in the studied animals by the higher prevalence of novel blood vasculature present in endometriosis compared with the endometrium inside the uterus (eutopic) and with non-angiogenic control tissues.

### Translation to human pathology

Despite MRI has proven a good diagnostic power for deeply invasive and ovarian endometriosis [13], [32] and in the recent decade protocols have been optimised to improve its diagnostic efficiency for these forms of endometriosis [33]–[35], peritoneal implants, especially if superficial, cannot be diagnosed readily by MRI [14], [15], [25].

The presence of strong angiogenic activity has been confirmed in human endometriosis [1], where over 80% of the blood vessels are pericyte-free. This exceeds the prevalence of new vessels in the eutopic endometrium [36]. Therefore, the enhancing properties of gadofosveset-trisodium could be helpful to detect endometriosis-associated angiogenesis in humans. Since the disease model used in this study resembles the superficial implants seen in women (uterine fragments are sutured to the peritoneal cavity or subcutaneously and do not deeply infiltrate other organs), our results suggest that gadofosveset-trisodium enhanced MRI has the potential to diagnose early endometriosis, including peritoneal implants in humans.

It may be envisaged that distinct kinds of peritoneal implants will be diagnosed with different sensitivities. For instance, red peritoneal lesions, typically with high angiogenic activity [1], may be diagnosed easier than black lesions (characterised by some extent of pericyte deposition on blood vessels) [1] and that the non-angiogenic white lesions may not be easily diagnosed. However, it should be also noted that in a clinical perspective, the highly angiogenic implants (especially the red ones) are responsible for the most important symptoms in patients. In addition, the most significant contribution of such non-invasive diagnostic tool will be the diagnosis of endometriosis in adolescent and in case of disease recurrence after treatment. These conditions are expected to be characterised by the presence of newly formed and highly angiogenic implants.

Gadofosveset-trisodium is clinically approved in several countries including the US and the EU, has a high tolerability and safety profile [26]–[28]. The use of this contrast agent and its diagnostic properties with regard to detecting endometriosis should now be tested in human settings to optimise an efficient protocol.

### Conclusions and possible benefits for the clinical management of endometriosis

The inability to diagnose non-invasively endometriosis is a well-recognised clinical issue, because as a consequence, the correct diagnosis is delayed. Such situation has important implications on the costs for the health-care system, on the quality of the private and professional life of the patients and can compromise the fertility and the functionality of peritoneal organs [2]–[4], [11], [12], [16].

A non-invasive diagnostic test for endometriosis would therefore alleviate all these conditions by allowing an early non-invasive diagnosis (especially important in young/adolescent women) and would avoid the costs and the risks of laparoscopy. Additionally, it would be of great utility for physicians by helping the selection of patients needing further invasive evaluation/treatment; helping plan adequately the operation, essential for treatment success; and helping distinguish recurrent disease from post-operative fibrosis, which presents similar symptoms as endometriosis.

## Materials and Methods

### Mice and surgery

Female Swiss mice were purchased from Harlan (Venray, The Netherlands) and housed with free access to water and food at the Central Animal Facilities of Maastricht University. Endometriosis was surgically induced in nine mice by autologous transplantation as described before [29]. In short, at 6–8 weeks of age, mice were anaesthetised using isoflurane (Forane, Abbott laboratories Ltd., UK; 5% and 2% in O_2_ for anaesthesia induction and maintenance, respectively), the abdomen was shaved and disinfected with iodine before covering with sterile drapes. A midline laparotomy was performed and the left uterine horn ligated proximally and distally with polyglactin 5×0 sutures, approximately 1.0 cm apart. Thereafter, the horn segment in between was resected, opened along its long axis and subsequently divided in equal halves by cutting perpendicularly to the long axis. Two uterine fragments of approximately 2–3 mm^2^ were prepared and were fixated using two polypropylene 6×0 sutures on opposite sites of the abdominal midline in the same mouse. One specimen was fixated to the peritoneal side of the abdominal wall with the endoluminal side facing intraperitoneally. The other specimen was fixated to the abdominal wall in a subcutaneous pocket with the endoluminal side towards the abdominal wall. Finally, the abdomen was closed with a running polyglactin 5×0 suture for the peritoneum, fascia and musculature, and a running intracutaneous poliglecaprone 5×0 suture for the skin.

### Histology and immunohistochemistry

At sacrifice, induced endometriotic lesions with surrounding tissues and uterus were excised and the size of the lesions was assessed with a calliper. Subsequently, tissues were fixed in buffered formalin, embedded in paraffin and cut in 5 μm thick sections. For histology, tissues were stained with haematoxylin and eosin. Immunohistochemistry for cluster of differentiation 31 (CD31; Entrez Gene: PECAM-1, platelet/endothelial cell adhesion molecule) was performed using the rat anti-mouse antibody clone MEC13.3 (1:100; BD Pharmingen, Breda, The Netherlands) and proteinase K as antigen retrieval method. Sections were blocked with 2% rabbit serum, 2% BSA prior to antibody application. Biotinylated rabbit anti-rat secondary antibody (1:500; Jackson Immuno Research Laboratories, Inc., PA, USA) and avidin-biotin-HRP complex (Dako, Carpinteria, CA, USA) were subsequently used. For detection of pericytes, α-smooth-muscle-actin (aSMA; Entrez Gene: ACTA2 actin, alpha 2, smooth muscle, aorta) was employed as a marker. Heat-induced antigen retrieval in citrate buffer was used, followed by incubation with monoclonal anti-aSMA FITC-labelled antibody (1:3000; Sigma-Aldrich Chemie BV, Zwijndrecht, The Netherlands) and by anti-FITC-HRP conjugated secondary antibody (1:600, Roche Diagnostics Co). Diaminobenzidine solution (Dako, Glostrup, Denmark) was used as a chromogen.

### Vessel density quantification

Microscopy pictures (20X magnification) were taken in the different tissues in each of the six mice used for quantifications. For each single tissue 6–12 images were taken for CD31 and equal number of images for aSMA. Vessel quantification was further determined on the digital images with aid of the ImageJ program (National Institute of health, USA; http://rsb.info.nih.gov/ij/). Vessel number/pixel (1 pixel = 0.1 µm) was determined in each image by counting the CD31 positive and aSMA positive vessels. Afterwards the average among values obtained from the 6–12 images in each tissue in one mouse was computed to yield the vessel densities in the various tissues in each mouse.

These final vessel density values were further analysed in Figure 6. As described in the result section, in Figure 6A the CD31 positive vessel density in each tissue was averaged among the six mice used for quantification. The same was done for the aSMA positive vessel density. In Figure 6B, first the ratio CD31/aSMA positive blood vessels was computed for the various tissues in each mouse (endometriosis, uterus, peri-endometriosis, muscle). Subsequently, the ratios for each tissue were averaged among the six mice.

### Gadofosveset-trisodium

Gadofosveset-trisodium (C_33_H_40_GdN_3_Na_3_O_15_P; trisodium-{(2-(R)-[(4,4-diphenylcyclohexyl)hosphonooxymethyl]diethylenetriaminepentaacetato) (aquo) gadolinium(III), molecular weight of 975.88 g/mol) was purchased from Bayer AG (Leverkusen, Germany) under the commercial name Vasovist^TM^, at a stock concentration of 0.25 mMol/ml. For mouse-injection, gadofosveset-trisodium was diluted to 18 mM in saline.

### MRI

Mice were put under general anaesthesia with 5% isoflurane (Forane, Abbott laboratories Ltd., UK) in O_2_. Anaesthesia was maintained with 2% isoflurane in O_2_ during the whole MRI procedure using a breathing mask. Hearth and breath rhythm were monitored during the whole procedure.

For injecting the contrast agent, an insulin-needle was inserted into the tail-vein and connected through a 0.61 mm diameter (0,28 mm internal diameter) Portex® polythene tube (Smiths Medical International, Ltd, Kent, UK) to a syringe. The tube, 25 cm long, was necessary to operate the contrast agent injection while the mouse was positioned inside the scanner. Gadofosveset-trisodium (50 μL of a 18 mM solution) was injected to a final concentration of 0.03 mMol/Kg (mouse average body weight - mean±SD - 29±2 g), followed by saline injection to flush the contrast agent through the tube and vein.

Imaging was performed on a 7-Tesla dedicated animal MRI unit (Bruker Biospec 70/30 USR, BGA12S gradient system, Paravision 5.03, Bruker Biospin GmbH Ettlingen, Germany). Images were acquired with a quadrature volume resonator with a 72-mm inner diameter. First, for anatomical reference a set of T2 and T1 weighted images were obtained prior to contrast agent administration. The acquisition images for the T2-weighted scan were: 3D TurboRARE spin echo sequence, echo time (TE) 48 ms, repetition time (TR) 1500 ms, and acceleration (RARE-) factor 16. The T1 weighted image was also based on a 3D RARE spin echo pulse sequence: TE 5.4 ms, TR 500 ms, and RARE-factor 4. Both images were acquired at the same position with identical geometry. The acquisition matrix 150×150×30 and the voxel sizes were 0.2×0.2×0.5 mm for both scans.

Second, angiogenesis sensitive imaging was performed by DCE-MRI, which was based on multi-slice fast gradient echo sequence [21]–[23]. The sequence properties were TE 1.75 ms, TR 83 ms, flip angle 35°, 75 volume repetitions, matrix size of 150×150, pixel size 0.2×02 mm, 15 slices, 1-mm contiguous slice thickness. After a period of 90 seconds (approximately 4 repetitions) the contrast agent was injected within five seconds. In this procedure, the low-resolution MR images were serially acquired for 20 minutes after contrast agent injection. After DCE-MRI the above described anatomical T1 and T2 weighted scans were repeated with the same receiver amplifier gain settings to facilitate image subtraction.

### Image analysis

To quantify the dynamics of the contrast agent enhancement, the area-under-curve values were determined at different characteristic stages of the signal intensity curves (Figure 4B) in various regions of interest: (i) the area (denoted by ‘I’ from inwash) from the contrast agent arrival till the peak enhancement; (ii) the area (denoted by ‘C’ from circulation phase) from peak enhancement till 10 minutes after contrast injection; and (iii) the area (denoted by ‘O’ from outwash) from 10 minutes till 20 minutes after contrast agent injection. Characterisation of tissue enhancement was quantified by the ratio O/C.

### Ethical Approval

The experimental protocol complied with the Dutch Animal Experimentation Act and was approved by the Committee for Animal Welfare of the local institution. The current investigation conforms to the *Guide for the Care and Use of Laboratory Animals* published by the US National Institutes of Health (NIH Publication No. 85–23, revised 1996).

Local institution: Maastricht University, Faculty of Health, Medicine and Life Sciences. Review board: ‘Dier Experimenten Commissie’ (Experimental Animal Commission) from the ‘Centrale Proefdier Voorzieningen’ (central experimental animal services; http://www.cpv.unimaas.nl/). Protocol ID: DEC NR: 2009-175.
